# Review and analysis of clinical trials of selective RET inhibitors for the treatment of thyroid cancer

**DOI:** 10.3389/fonc.2025.1683624

**Published:** 2025-11-06

**Authors:** Yulu Zhou, Junjie Cao, Siqi Zhang, Mingjun Sun, Yaqi Zhang, Songzhe Li, Zining Luo, Pengyu Wang, Jiebin Xie

**Affiliations:** 1 Department of Gastrointestinal Surgery, Affiliated Hospital of North Sichuan Medical College, Nanchong, China; 2 North Sichuan Medical University, Nanchong, Sichuan, China; 3 Affiliated Hospital of North Sichuan Medical University, Nanchong, Sichuan, China; 4 Independent Researcher, Xianning, China

**Keywords:** selective RET inhibitors, thyroid cancer, selpercatinib, pralsetinib, RET mutations

## Abstract

The global incidence of thyroid cancer has increased significantly, and patients with advanced, recurrent, or radioiodine-refractory disease face a severe shortage of effective treatment options. The *RET* proto-oncogene serves as a key driver in the development of thyroid cancer, and its alterations are closely associated with highly aggressive tumor subtypes. Although the emergence of highly selective RET inhibitors (such as selpercatinib and pralsetinib) has revolutionized the treatment landscape, their complete clinical development pathway, rational combination strategies, and future research priorities still require systematic clarification. Understanding the development trends of these drugs is important for guiding clinical decision-making, optimizing trial design, and accelerating new drug development. We searched 16 clinical trial registries, and identified 18 studies registered up to 21 March 2025. Our analysis revealed that among the 18 eligible trials, the majority were Phase 1/2 studies. Selpercatinib and pralsetinib are the most frequently studied RET inhibitors. Notably, research on next-generation RET inhibitors as monotherapy approaches (e.g., LOXO-260, enbezotinib, SY-5007 and TY-1091) is currently underway. Additionally, combination regimens incorporating these inhibitors with agents such as ¹³¹I, recombinant human thyroid-stimulating hormone (rhTSH), and anti-programmed cell death protein 1 (anti-PD-1) antibodies are becoming an increasingly important area of investigation. While selective RET inhibitors have demonstrated therapeutic potential, concerns regarding drug resistance and toxicity persist. Therefore, future strategies should prioritize the development of next-generation inhibitors and the optimization of combination regimens to improve outcomes for *RET*-altered thyroid cancer patients.

## Introduction

Thyroid cancer is the most common malignant tumor of the endocrine system, and its global incidence rate continues to rise significantly. It ranked as the seventh most common cancer globally in 2024 and is projected to rank fourth worldwide in the future (1–3). Although thyroid cancer generally has a favorable prognosis, its management faces significant limitations. For patients with advanced, radioiodine-refractory differentiated thyroid cancer (DTC) and medullary thyroid cancer (MTC) harboring specific genetic alterations such as RET mutations, conventional therapies yield suboptimal outcomes. Surgical intervention is often inadequate for eradicating metastatic disease (4), whereas radioiodine therapy fails due to tumor cell dedifferentiation, ultimately necessitating a shift to palliative treatment goals. However, the clinical application of these approved multi-kinase inhibitors (e.g., sorafenib, lenvatinib, vandetanib, and cabozantinib) is significantly constrained by off-target effects. First, the inhibition of targets like VEGFR causes substantial dose-limiting toxicities, often leading to treatment discontinuation. Furthermore, their pharmacodynamic action against VEGFR is stronger than against RET, and they show insufficient activity against key resistance mutations such as *RET V804*. These limitations ultimately restrict their overall efficacy (5).

A comprehensive analysis of the molecular mechanisms underlying thyroid cancer revealed that the *RET* proto-oncogene is a central driver of its development. *RET* gene rearrangements and somatic mutations are common genetic events in thyroid cancer and are closely associated with tumor development and exhibit characteristic distributions: approximately 25% of hereditary MTC cases harbor *RET* germline activating mutations (associated with MEN 2A/B), and approximately 45% of sporadic MTC cases carry *RET* somatic mutations. In differentiated thyroid cancer, *RET* gene fusions account for approximately 6–10% of papillary thyroid cancer (PTC) cases and 6% of poorly differentiated thyroid cancer (PDTC) cases but are rare in anaplastic thyroid cancer (ATC) cases. The prevalence of *RET* fusions is greater in radiation-induced thyroid cancer (approximately 60–80%) (6–11). These high-frequency and characteristic *RET* mutations clearly indicate their key value as therapeutic targets. Abnormal activation of RET (via mechanisms such as gene rearrangements or point mutations) can activate key downstream signaling pathways such as the RAS/MAPK and PI3K/AKT pathways, driving tumor cell proliferation, survival, invasion, angiogenesis, and metastasis (12). Clinical data indicate that, compared with *RAS*-mutated patients, *RET*-driven tumors are more aggressive; more likely to exhibit extrathyroid extension, multifocality, and distant metastasis; and a poorer prognosis (13). The central oncogenic role of RET in thyroid cancer, its association with unfavorable prognosis, and the limitations of multi-kinase inhibitor therapy collectively establish a powerful rationale for drug development. This, in turn, creates a compelling clinical need for highly selective and potent RET inhibitors.

In contrast to multi-kinase inhibitors, selective RET inhibitors achieve high-specificity binding to the RET kinase domain, competitively occupying the ATP-binding pocket, thereby effectively inhibiting RET autophosphorylation and its downstream signaling pathways. This mechanism enables precise blockade of oncogenic RET signaling, demonstrating potent inhibitory activity against a range of *RET* alterations from the M918T point mutation and *CCDC6-RET* fusion to the multi-kinase inhibitor-resistant *RET V804* gatekeeper mutation (14). The LIBRETTO-001 trial evaluated selpercatinib in *RET*-mutated MTC patients without prior vandetanib or cabozantinib. Selpercatinib was associated with a higher objective response rate (ORR) (73% vs 69%) and longer progression-free survival (PFS) (1-year PFS 92% vs 82%) compared to the prior standard of vandetanib or cabozantinib. In the NCT03037385 clinical trial, pralsetinib achieved an ORR of 71% in patients with *RET*-mutated MTC and an ORR of 89% in patients with *RET* fusion-positive thyroid cancer. This highly specific targeting not only significantly improves efficacy but also greatly improves patient tolerance (15). These drugs were approved by the U.S. Food and Drug Administration (FDA) in 2020 for second-line treatment of advanced MTC (16, 17).

However, the clinical application of selective RET inhibitors continues to face significant challenges. First, the rate of complete response (CR) remains very low, and these agents are unable to cure the disease (14). Second, resistance mechanisms are complex, with on-target mutations (such as the *G810* series) and bypass activation (e.g., *KRAS/MET*) leading to disease progression in the majority of patients (18, 19). Third, potential long-term risks exist; animal studies suggest possible impacts on bone/tooth development and neurological function, though the specific implications for human fertility, childhood growth and development, and neurobiology remain to be fully elucidated (20). While these advances have brought a paradigm shift in improving patient survival, they also introduce new challenges and, in parallel, new opportunities. Addressing these will require rigorously evaluating optimal dosing regimens, defining precise indications, and establishing long-term safety and efficacy data.

## Methods

**Figure 1:**
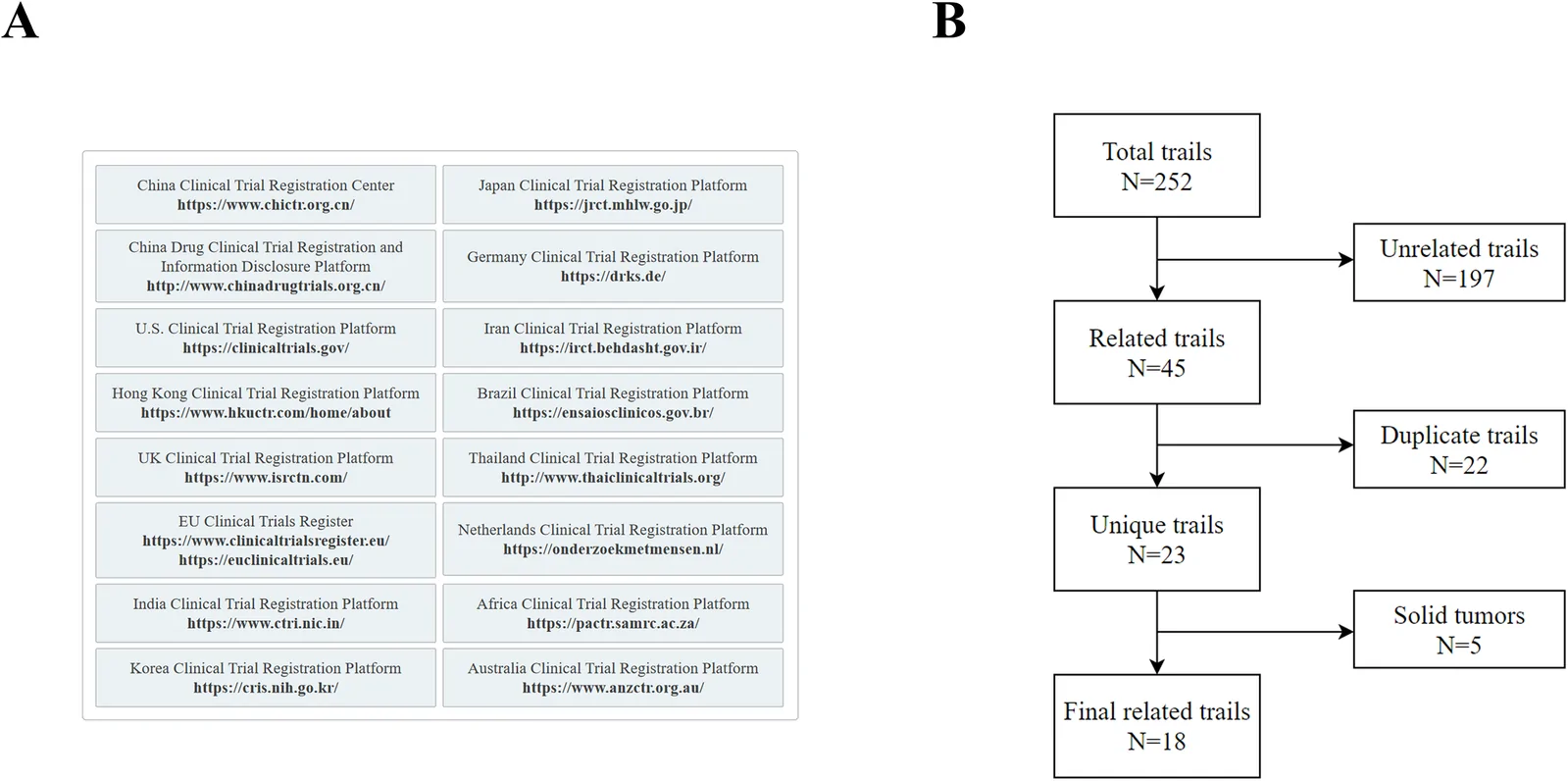
Clinical trial sources and inclusion and exclusion flowchart.

This study aims to systematically evaluate the clinical research landscape of selective RET inhibitors in the treatment of thyroid cancer. We systematically searched 16 global clinical trial registries up to March 21, 2025. Our strategy aimed to cover representative registries across six continents, thereby maximizing retrieval comprehensiveness and minimizing publication bias. The core search encompassed primary registries recognized by the World Health Organization’s International Clinical Trials Registry Platform (WHO ICTRP), such as ClinicalTrials.gov. To increase the coverage of trial information from key regions, we supplemented the search with regional official platforms, including China’s National Medical Products Administration (NMPA) Drug Clinical Trial Registration and Information Disclosure Platform, the Hong Kong Clinical Trials Register, and the African Clinical Trial Registry, among others (**Figure 1A**). The inclusion of all registries was based on the objective criteria of “public accessibility” and “data availability”.

The search was conducted via the core keywords “thyroid cancer” and “selective RET inhibitors” via the Medical Subject Headings (MeSH) in PubMed/MEDLINE and the Emtree thesaurus in Embase. The initial search identified 252 candidate studies.

Study screening was performed independently by two investigators (Zhou and Cao) according to the following procedure (**Figure 1B**):

(1) The screening of studies was based on the title, interventions, and medical conditions; those clearly not meeting the inclusion criteria were excluded. The inclusion criteria were defined as clinical studies involving thyroid cancer and evaluating RET-targeted therapy. The exclusion criteria comprised studies unrelated to thyroid cancer or those not involving RET-targeted therapy. Additionally, due to potential inconsistencies or incomplete recording of trial identifiers (e.g., NCT numbers) across different databases or registries, it was difficult to directly and accurately identify and merge duplicate records at this preliminary screening stage.

(2) For studies that passed the initial screening, duplicate records were identified and removed by comparing detailed information such as trial registration numbers, study design, and patient baseline characteristics.

(3) The full registry entries of the remaining unique studies were reviewed to confirm eligibility. At this stage, pancancer studies that did not explicitly include a thyroid cancer patient subgroup or for which a subgroup analysis was planned were excluded.

Any disagreements between the two investigators were resolved through discussion; if a consensus could not be reached, a third investigator (Zhang) arbitrated.

To ensure a comprehensive overview of the clinical research landscape, our search strategy incorporated not only conventional trials but also expanded access programs and studies with unusual statuses (e.g., withdrawn). Recognizing the distinct nature and evidence levels of these study types, we established a clear *a priori* classification framework to balance comprehensiveness with methodological rigor. The primary dataset was strictly limited to conventional prospective interventional clinical trials, which constitute the core of hypothesis-testing research, thereby allowing our analysis to focus on studies with the highest methodological evidence. Furthermore, expanded access programs, which serve as a critical bridge between clinical development and real-world application, were categorized into a supplementary dataset (**Supplementary Table 1**). Withdrawn studies with zero enrollment were also included in this supplementary category. Importantly, this supplementary category was explicitly restricted to the specific types mentioned above, which were sourced from clinical trial registries. Other nonprospective designs, such as retrospective observational studies, were excluded from this study.

Data extraction and management for the final included studies was performed using Microsoft Excel. The world map was generated with the ggplot2 package in R software (version 4.5.1), and all other graphics were created using Origin 2024.

## Results

A total of 18 studies were ultimately included in this analysis. On the basis of predefined criteria, 14 studies constituted the primary dataset, and all subsequent analyses concerning trial phase, status, and design were based on this dataset. The remaining four studies (comprising three expanded access programs and one withdrawn study with zero participants) were included in the supplementary dataset.

**Figure 2:**
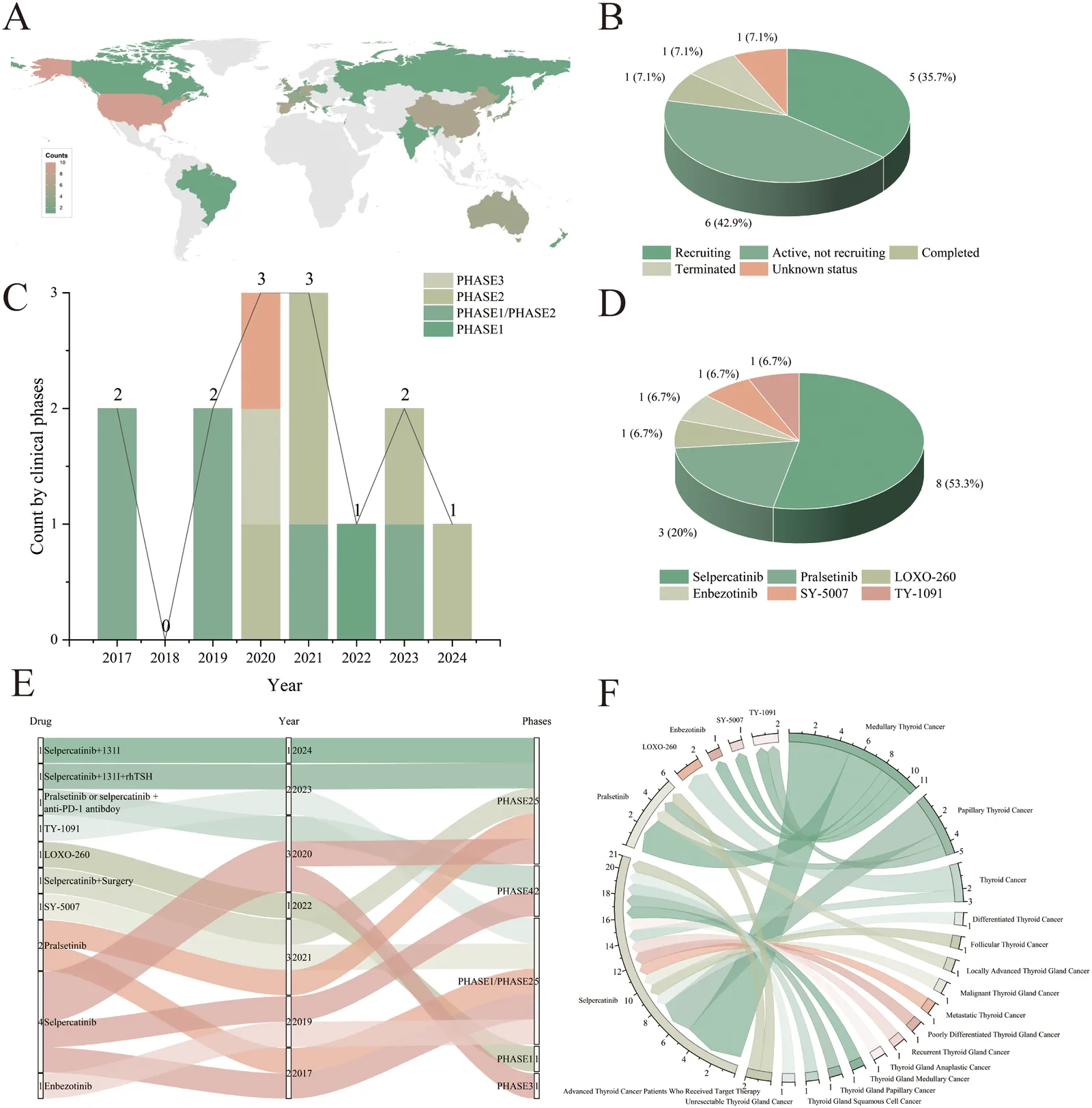
Comprehensive analysis of clinical trials of selective RET inhibitors for thyroid cancer treatment. **(A)** Distribution of selective RET inhibitors for the treatment of thyroid cancer by country/region; **(B)** Distribution of the current status of clinical trials in the primary dataset; **(C)** Registration timeline of selective RET inhibitor trials in the primary dataset, stratified by clinical phase; **(D)** Distribution of different selective RET inhibitors in the primary dataset; **(E)** Distribution of clinical trial phases across the different selective RET inhibitors in the primary dataset; **(F)** Distribution of different selective RET inhibitors for the treatment of thyroid cancer types.

Geographically, the distribution of all studies (including both the primary and supplementary datasets) was concentrated primarily in the United States, the European Union, and East Asia (**Figure 2A**). The status distribution within the primary dataset revealed that ‘Recruiting’ (n = 5, 35.7%) and ‘Active, not recruiting’ (n = 6, 42.9%) were the most prevalent phases. The presence of one ‘Completed’ trial (n = 1) indicates the availability of initial results, whereas the combined 14.2% of studies that were ‘Terminated’ or had an ‘Unknown status’ highlight challenges in drug development and implementation (**Figure 2B**).

Statistical analyses revealed that all studies in the primary dataset were conducted between 2017 and 2024. Among these, the majority were Phase 2 studies (n = 11), followed by Phase 1 studies (n = 7) (**Figure 2C**). The most commonly used selective RET inhibitor for the treatment of thyroid cancer is selpercatinib (n = 8, including drug combinations hereafter), followed by pralsetinib (n = 3). Additionally, the drugs LOXO-260, enbezotinib, SY-5007 and TY-1091 each appeared once in the studies (**Figure 2D**). The supplementary dataset comprised studies conducted from 2019 to 2023, that exclusively involved selpercatinib, pralsetinib, and LOXO-260 (**Supplementary Table 1**).

Within the primary dataset, selpercatinib and pralsetinib are used in multiple trial phases, including Phases 1, 2, 3, and 4. Enbezotinib, LOXO-260, SY-5007, and TY-1091, which appeared primarily in Phase 1 and 2 trials starting in 2019, appear to be in the early stages of development and are currently undergoing preliminary safety and efficacy assessments (**Figure 2E**). Since 2020, there has been an increase in the number of studies of selective RET inhibitors in combination with other drugs; all of these are in early phases (Phase 1, Phase 2), including combinations with ¹³¹I, recombinant human thyroid-stimulating hormone (rhTSH), and anti-programmed cell death protein 1 (PD-1) antibodies. Notably, selpercatinib monotherapy was used only until 2020 (n = 5, excluding post-drug surgery) (**Figure 2E**). In contrast, all interventional studies in the supplementary dataset involved monotherapy regimens (**Supplementary Table 1**).

Analysis by thyroid cancer type across all studies (including both primary and supplementary datasets) revealed a greater focus on medullary thyroid cancer (MTC) (n = 11) for selective RET inhibitors. Notably, selpercatinib is widely used in studies for the treatment of multiple types of thyroid cancer (**Figure 2F**).

## Discussion

The emergence of selective RET inhibitors has led to revolutionary breakthroughs in the treatment of thyroid cancer. Through a systematic analysis of 18 clinical trials, this study revealed that the field is currently in a critical phase of transition from early exploration to clinical application. Currently, global R&D efforts exhibit significant regional disparities. Western countries are leading this effort, benefiting from well-established clinical research systems, high rates of genetic testing, and robust policy support. In contrast, regions such as Southeast Asia and Africa lag behind, primarily due to insufficient medical resources and outdated regulatory frameworks. Notably, given the high incidence of *RET* rearrangement mutations in medullary thyroid cancer (MTC), existing research has focused primarily on this specific subtype.

Among existing drugs, selpercatinib and pralsetinib, as representative agents, have undergone systematic clinical evaluations from Phase 1 to Phase 4, demonstrating significant clinical value (**Supplementary Table 2**). However, as their application has intensified, the limitations of these drugs have gradually become apparent. In terms of safety, the ARROW global trial (NCT03037385) and the Selpercatinib Phase 1/2 study (NCT03157128) reported adverse reactions associated with these drugs, including hepatotoxicity, cardiovascular events, and pulmonary complications (21, 22). More concerning is the increasingly evident phenomenon of clinical drug resistance. This primarily manifests in two ways: reduced drug binding affinity due to *RET* G810R/S/C mutations, and activation of bypass signaling pathways triggered by *MET* amplification. These mechanisms collectively lead to diminished treatment efficacy and accelerated disease progression (23–25).

To overcome these treatment bottlenecks, the development of a new generation of RET inhibitors is accelerating globally. Since 2019, several innovative drugs, including enbezotinib, LOXO-260, SY-5007, TY-1091, and HA121-28, have entered clinical research phases (**Supplementary Table 2**). Among these, SY-5007 stands out. It not only has manageable adverse reactions and lower doses, but also demonstrates lower discontinuation and mortality rates (23.8%, 1.6%, and 0%, respectively) compared to selpercatinib and pralsetinib. It achieved ORR of 52.2% and 42.9% for *RET*-mutated MTC and *RET*-fusion-positive papillary thyroid cancer (PTC), respectively (26). Although enbezotinib was discontinued because of an unfavorable risk–benefit assessment (27), other candidate drugs remain in active clinical evaluation.

Combination therapy strategies demonstrate significant potential for development. The combination of selpercatinib and crizotinib has achieved successful outcomes in overcoming drug resistance in the treatment of non-small cell lung cancer, providing an important reference for combination therapy in thyroid cancer patients with *RET* mutations (28). Notably, studies have shown that RET inhibitors can enhance the efficacy of immunotherapy by regulating the tumor microenvironment (29). This finding lays a solid theoretical foundation for combining RET inhibitors with immunotherapies such as PD-1 inhibitors. Currently, multiple Phase 1/2 clinical trials are underway globally to systematically evaluate the safety and efficacy of different combination therapy regimens (traditional combination, immunotherapy combination, and radiotherapy enhancement).

In the future, the field should focus on several key areas: developing next-generation inhibitors against drug-resistant mutations; optimizing combination regimens, including with immunotherapy; and expanding research into *RET*-altered thyroid cancer subtypes. Additionally, efforts must strengthen the accumulation of real-world evidence and promote a more balanced allocation of global R&D resources. Despite ongoing challenges, as research deepens and treatment strategies continue to be optimized, selective RET inhibitors hold promise to drive *RET*-altered thyroid cancer treatment into a new era of greater precision and efficacy, ultimately resulting in improved treatment outcomes and quality of life for patients.

## Data Availability

The original contributions presented in the study are included in the article/**Supplementary Material**. Further inquiries can be directed to the corresponding authors.

## Supplementary material

Supplementary Table 1 Clinical characteristics of select RET inhibitors for the treatment of thyroid cancer.

Supplementary Table 2 Summary of the targets, advantages, and challenges of different selective RET Inhibitors.
