# Correction: Review and analysis of clinical trials of selective RET inhibitors for the treatment of thyroid cancer

**DOI:** 10.3389/fonc.2026.1939348

**Published:** 2026-08-14

**Authors:** Yulu Zhou, Junjie Cao, Siqi Zhang, Mingjun Sun, Yaqi Zhang, Songzhe Li, Zining Luo, Pengyu Wang, Jiebin Xie

**Affiliations:** 1 Department of Gastrointestinal Surgery, Affiliated Hospital of North Sichuan Medical College, Nanchong, China; 2 North Sichuan Medical University, Nanchong, Sichuan, China; 3 Affiliated Hospital of North Sichuan Medical University, Nanchong, Sichuan, China; 4 Independent Researcher, Xianning, China

**Keywords:** selective RET inhibitors, thyroid cancer, selpercatinib, pralsetinib, RET mutations

Affiliation “School of Engineering, China Pharmaceutical University, Nanjing, China” was erroneously included in the paper. This affiliation has now been removed for author Pengyu Wang.

“Independent Researcher, Xianning, China” was omitted from the paper. This affiliation has now been added for author Pengyu Wang.

The original version of this article has been updated.

