# Supplementary material for: Review and analysis of clinical trials of selective RET inhibitors for the treatment of thyroid cancer
Source: Front Oncol. 2025 Nov 6;15:1683624. doi: 10.3389/fonc.2025.1683624 (PMC12631409; doi:10.3389/fonc.2025.1683624)
Supplement: Supplementary Table 1 — Clinical characteristics of select RET inhibitors for the treatment of thyroid cancer. [file Table1.docx]

****Supplementary Materials****

**Supplementary Table 1: Clinical characteristics of select RET inhibitors for the treatment of thyroid cancer**

| NCT Number | Study URL | Sex | Age | Phases | Enrollment | Study Type | Interventions | Start year |
| --- | --- | --- | --- | --- | --- | --- | --- | --- |
| NCT06458036 | https://clinicaltrials.gov/study/NCT06458036 | ALL | CHILD, ADULT | Phase2 | 13 | INTERVENTIONAL | Selpercatinib+131I | 2024 |
| NCT05668962 | https://clinicaltrials.gov/study/NCT05668962 | ALL | CHILD, ADULT, OLDER_ADULT | Phase2 | 30 | INTERVENTIONAL | Selpercatinib+131I+rhTSH | 2023 |
| NCT04759911 | https://clinicaltrials.gov/study/NCT04759911 | ALL | CHILD, ADULT, OLDER_ADULT | Phase2 | 30 | INTERVENTIONAL | Selpercatinib+surgery | 2021 |
| NCT06195228 | https://clinicaltrials.gov/study/NCT06195228 | ALL | ADULT, OLDER_ADULT | Phase4 | 200 | INTERVENTIONAL | Pralsetinib or selpercatinib with or without anti-PD-1 antibody | 2020 |
| NCT03037385 | https://clinicaltrials.gov/study/NCT03037385 | ALL | ADULT, OLDER_ADULT | Phase1\|Phase2 | 589 | INTERVENTIONAL | Pralsetinib | 2017 |
| NCT03157128 | https://clinicaltrials.gov/study/NCT03157128 | ALL | CHILD, ADULT, OLDER_ADULT | Phase1\|Phase2 | 856 | INTERVENTIONAL | Selpercatinib | 2017 |
| NCT03899792 | https://clinicaltrials.gov/study/NCT03899792 | ALL | CHILD, ADULT | Phase1\|Phase2 | 50 | INTERVENTIONAL | Selpercatinib | 2019 |
| NCT04161391 | https://clinicaltrials.gov/study/NCT04161391 | ALL | ADULT, OLDER_ADULT | Phase1\|Phase2 | 41 | INTERVENTIONAL | Enbezotinib | 2019 |
| NCT04211337 | https://clinicaltrials.gov/study/NCT04211337 | ALL | CHILD, ADULT, OLDER_ADULT | Phase3 | 291 | INTERVENTIONAL | Selpercatinib | 2020 |
| NCT04280081 | https://clinicaltrials.gov/study/NCT04280081 | ALL | ADULT, OLDER_ADULT | Phase2 | 77 | INTERVENTIONAL | Selpercatinib | 2020 |
| NCT05241834 | https://clinicaltrials.gov/study/NCT05241834 | ALL | CHILD, ADULT, OLDER_ADULT | Phase1 | 110 | INTERVENTIONAL | LOXO - 260 | 2022 |
| NCT05278364 | https://clinicaltrials.gov/study/NCT05278364 | ALL | ADULT, OLDER_ADULT | Phase1\|Phase2 | 184 | INTERVENTIONAL | SY-5007 | 2021 |
| NCT05675605 | https://clinicaltrials.gov/study/NCT05675605 | ALL | ADULT, OLDER_ADULT | Phase1\|Phase2 | 248 | INTERVENTIONAL | TY-1091 | 2023 |
| NCT06482086 | https://clinicaltrials.gov/study/NCT06482086 | ALL | ADULT, OLDER_ADULT | Phase2 | 75 | INTERVENTIONAL | Pralsetinib | 2021 |
| NCT03906331 | https://clinicaltrials.gov/study/NCT03906331 | ALL | ADULT, OLDER_ADULT |  |  | EXPANDED_ACCESS | Selpercatinib | 2019 |
| NCT05225259 | https://clinicaltrials.gov/study/NCT05225259 | ALL | CHILD, ADULT, OLDER_ADULT |  |  | EXPANDED_ACCESS | LOXO - 260 | 2022 |
| NCT04204928 | https://clinicaltrials.gov/study/NCT04204928 | ALL | CHILD, ADULT, OLDER_ADULT |  |  | EXPANDED_ACCESS | Pralsetinib | 2019 |
| NCT04760288 | https://clinicaltrials.gov/study/NCT04760288 | ALL | CHILD, ADULT, OLDER_ADULT | Phase3 | 0 | INTERVENTIONAL | Pralsetinib | 2023 |
|  |  |  |  |  |  |  |  |  |

**Supplementary Table 2: Summary of the targets, advantages, and challenges of different selective RET Inhibitors.**

| **Selective RET Inhibitors** | **Target(s)** | **Key Advantages** | **Key Challenges** | **Key Clinical Trial** | **Trial Phase** |
| --- | --- | --- | --- | --- | --- |
| Selpercatinib（LOXO-292）[1] | RET (Receptor Tyrosine Kinase), which includes *RET* mutations (e.g., M918T, V804, etc.) and *RET* fusions (e.g., *CCDC6-RET*, *NCOA4-RET*, etc.) | 1. It has pioneered the era of RET precision therapy, with efficacy far superior to that of multi-kinase inhibitors. 2. It demonstrates high response rates and durable efficacy in both treatment-naive and pretreated patients. 3. It exhibits favorable brain-penetrant activity. | The main occurrence is target-based drug resistance, particularly *RET* solvent-front mutations (e.g., *G810X*), which limits its long-term efficacy. | LIBRETTO-001（NCT03157128） | Phase1\|Phase2 |
| pralsetinib(BLU-667)[2] | RET (Receptor Tyrosine Kinase), which includes *RET* mutations (e.g., M918T, V804, etc.) and *RET* fusions (e.g., *CCDC6-RET*, *NCOA4-RET*, etc.) | 1. Together with Selpercatinib, it has laid the foundation for precision therapy of *RET*-altered diseases, with remarkable efficacy. 2. It is administered orally once daily, offering convenient medication. 3. It is also effective against gatekeeper mutations such as *V804M/L*. | The main occurrence is target-based drug resistance, particularly *RET* solvent-front mutations (e.g., *G810X*), which limits its long-term efficacy. | ARROW (NCT03037385) | Phase1\|Phase2 |
| SY-5007[3] | RET (Receptor Tyrosine Kinase), which encompasses *RET* fusions (e.g., *KIF5B-RET*, *CCDC6-RET*) and *RET* mutations (e.g., M918T) | 1. It is a domestic innovative drug, and preliminary data show that its efficacy is comparable to that of first-generation drugs. 2. Activity has been observed in both treatment-naive and pretreated patients. 3. It has controllable safety and minimal off-target toxicity. | 1. Drug resistance issue: Preclinical data indicate that its drug resistance mechanism is mainly off-target; however, it is still necessary to monitor whether it will develop target-based resistance like first-generation drugs. 2. The maturity of its clinical data is lower than that of marketed drugs. | NCT05278364 | Phase1 |
| Enbezotinib(TPX-0046)[4] | RET (wild-type and multiple mutant variants, including the solvent-front mutation *G810R*); it does not inhibit VEGFR2 | 1. It is specifically designed to overcome drug resistance to first-generation agents, and is particularly potent against solvent-front mutations (*G810X*). 2. The dual targets (*RET/SRC*) may provide more comprehensive signal inhibition and the ability to overcome drug resistance. 3. It has a unique structure and no cross-resistance with first-generation drugs. | 1. Safety is unknown: Dual-target inhibition may lead to a unique or unknown adverse reaction profile, which requires clinical verification. 2. Its clinical development is in the early stage, and its efficacy remains to be confirmed. | NCT04161391 | Phase1\|Phase2 |
| LOXO - 260[5] | *RET* (including wild-type, drug-resistant mutations such as the solvent-front mutation *G810X* and gatekeeper mutations) | 1. Precisely targets drug-resistant mutations: Specifically designed to overcome acquired resistance to first-generation RET inhibitors (e.g., selpercatinib), with high specificity. 2. Conquers key drug-resistant sites: It can potently inhibit the most common drug resistance mechanisms—RET solvent-front mutations (*G810X*) and gatekeeper mutations—and remains effective even when these mutations occur alone or in combination. 3. High selectivity: Great emphasis is placed on high selectivity for the RET target in its design, aiming to minimize toxicity caused by off-target effects and achieve better potential safety profiles. | 1. Specifically designed for patients with drug resistance: Its positioning and advantages in treatment-naive patients remain unclear.2. 2. Its clinical development is in the earliest stage, and all relevant data are unknown. | NCT05241834 | Phase1 |
| TY-1091[6] | RET (wild-type and multiple drug-resistant mutations), including: • Solvent-front mutations: *G810S/R/C/V* • Gatekeeper mutations: *V804M/L/E* • Other secondary mutations: *Y806C/N*, *V728A* • Double mutations: *V804M/G810S*, *M918T/G810S* | 1. Broad-spectrum anti-mutation activity: It is a pan-*RET* mutation inhibitor, and its advantage lies in its ability to broadly inhibit a variety of RET drug-resistant mutations, including solvent-front mutations (*G810S/R/C/V*), gatekeeper mutations (*V804M/L/E*), and even complex double mutations (e.g., *V804M/G810S*). 2. Potent inhibitory effect: Preclinical data show that its half-maximal inhibitory concentration (IC₅₀) against various RET variants is extremely low, with strong potency—superior to the first-generation inhibitor cabozantinib, and equivalent to or even better than the potency of marketed second-generation inhibitors. 3. Addressing complex drug resistance: Its potent inhibition of double mutations indicates that it is expected to address more complex clinical drug resistance issues, providing potential for subsequent treatment. | 1. Earliest stage of research and development: It is still in the pure preclinical research stage currently, and all its promising potential has only been demonstrated in laboratory models, without any verification in humans—this constitutes the greatest uncertainty. 2. Risks hidden in "broad-spectrum" activity: As a "pan-*RET* mutation inhibitor," its broad-spectrum property may lead to unknown and more complex off-target toxicity, and its safety window awaits rigorous evaluation in Phase I clinical trials. 3. Challenges in druggability: Excellent preclinical data do not always translate into clinical success, and its performance in humans, such as pharmacokinetics and bioavailability, remains unknown. | NCT05675605 | Phase1\|Phase2 |

**References**

[1] Wirth LJ, Sherman E, Robinson B, Solomon B, Kang H, Lorch J, et al. Efficacy of Q23 selpercatinib in RET-altered thyroid cancers. N Engl J Med. (2020) 383:825–35. doi: 10.1056/NEJMoa2005651

[2] Subbiah V, Hu MI, Wirth LJ, Schuler M, Mansfield AS, Curigliano G, et al. Pralsetinib for patients with advanced or metastatic RET-altered thyroid cancer (ARROW): a multi-cohort, open-label, registrational, phase 1/2 study. Lancet Diabetes Endocrinol. (2021) 9:491–501. doi: 10.1016/S2213-8587(21)00120-0

[3] Li W, Wang Y, Xiong A, Gao G, Song Z, Zhang Y, et al. First-in-human, phase 1 dose-escalation and dose-expansion study of a RET inhibitor SY-5007 in patients with advanced RET-altered solid tumors. Signal Transduct Target Ther. (2024) 9:300. doi: 10.1038/s41392-024-02006-9

[4] Drilon AE, Zhai D, Rogers E, Deng W, Zhang X, Jung J, et al. The next-generation RET inhibitor TPX-0046 is active in drug-resistant and naïve RET-driven cancer models. J Clin Oncol. 2020;38 (15_suppl):3616-3616. doi:10.1200/JCO.2020.38.15_suppl.3616
[5] Pennell NA, Wirth LJ, Gainor JF, Rotow JK, Johnson ML, Bauer TM, et al. A first-in-human phase 1 study of the next-generation RET inhibitor, LOXO-260, in RET inhibitor refractory patients with RET-altered cancers (trial in progress). J Clin Oncol. 2022;40 (16_suppl):TPS8595-TPS8595. doi:10.1200/JCO.2022.40.16_suppl.TPS8595

[6] Niu C, Zheng M, Wang H, Ji K, Wang G, Ni R, et al. Abstract 3419: TY-1091, a highly selective and potent second-generation RET inhibitor, demonstrates superior antitumor activity in multiple RET-mutant models. Cancer Res. 2023;83(7_Supplement):3419-3419. doi:10.1158/1538-7445.AM2023-3419
